# Gene-expression profiles of pretreatment biopsies predict complete response of rectal cancer patients to preoperative chemoradiotherapy

**DOI:** 10.1038/s41416-022-01842-2

**Published:** 2022-05-21

**Authors:** Georg Emons, Noam Auslander, Peter Jo, Julia Kitz, Azadeh Azizian, Yue Hu, Clemens F. Hess, Claus Roedel, Ulrich Sax, Gabriela Salinas, Philipp Stroebel, Frank Kramer, Tim Beissbarth, Marian Grade, Michael Ghadimi, Eytan Ruppin, Thomas Ried, Jochen Gaedcke

**Affiliations:** 1 Section of Cancer Genomics, Genetics Branch, National Cancer Institute, National Institutes of Health, Bethesda, MD USA; 2 Department of General, Visceral and Pediatric Surgery, University Medical Center, Göttingen, Germany; 3 Cancer Data Science Laboratory, Center for Cancer Research, National Cancer Institute, National Institutes of Health, Bethesda, MD USA; 4 Program in Molecular and Cellular Oncogenesis, The Wistar Institute, Philadelphia, PA USA; 5 Department of Pathology, University Medical Center, Göttingen, Germany; 6 Department of Radiotherapy and Radio-oncology, University Medical Center, Göttingen, Germany; 7 Department of Radiation Oncology, University Hospital Johann Wolfgang Goethe University, Frankfurt, Germany; 8 Department of Medical Informatics, University Medical Center, Göttingen, Germany; 9 Transcriptome and Genome Analysis Laboratory (TAL), Department of Developmental Biochemistry, University of Göttingen, Göttingen, Germany; 10 Department of Medical Statistics, University Medical Center, Göttingen, Germany

**Keywords:** Radiotherapy, Predictive markers, Surgical oncology, Genetics research

## Abstract

**Purpose:**

Preoperative (neoadjuvant) chemoradiotherapy (CRT) and total mesorectal excision is the standard treatment for rectal cancer patients (UICC stage II/III). Up to one-third of patients treated with CRT achieve a pathological complete response (pCR). These patients could be spared from surgery and its associated morbidity and mortality, and assigned to a “watch and wait” strategy. However, reliably identifying pCR based on clinical or imaging parameters remains challenging.

**Experimental design:**

We generated gene-expression profiles of 175 patients with locally advanced rectal cancer enrolled in the CAO/ARO/AIO-94 and -04 trials. One hundred and sixty-one samples were used for building, training and validating a predictor of pCR using a machine learning algorithm. The performance of the classifier was validated in three independent cohorts, comprising 76 patients from (i) the CAO/ARO/AIO-94 and -04 trials (*n* = 14), (ii) a publicly available dataset (*n* = 38) and (iii) in 24 prospectively collected samples from the TransValid A trial.

**Results:**

A 21-transcript signature yielded the best classification of pCR in 161 patients (Sensitivity: 0.31; AUC: 0.81), when not allowing misclassification of non-complete-responders (False-positive rate = 0). The classifier remained robust when applied to three independent datasets (*n* = 76).

**Conclusion:**

The classifier can identify >1/3 of rectal cancer patients with a pCR while never classifying patients with an incomplete response as having pCR. Importantly, we could validate this finding in three independent datasets, including a prospectively collected cohort. Therefore, this classifier could help select rectal cancer patients for a “watch and wait” strategy.

**Translational relevance:**

Forgoing surgery with its associated side effects could be an option for rectal cancer patients if the prediction of a pathological complete response (pCR) after preoperative chemoradiotherapy would be possible. Based on gene-expression profiles of 161 patients a classifier was developed and validated in three independent datasets (*n* = 76), identifying over 1/3 of patients with pCR, while never misclassifying a non-complete-responder. Therefore, the classifier can identify patients suited for “watch and wait”.

## Introduction

Colorectal cancer is the third most common cancer in the United States (145,600 cases), of which approximately one-third are located in the rectum (SEER Cancer Stat Facts 2019). Rectal cancer has been treated primarily by surgery for over 200 years. Attributable to effective screening programs, the introduction of chemoradiotherapy (CRT), and improved surgical techniques [1], the mortality rate for rectal cancers has been significantly reduced [2, 3]. The current therapy for rectal cancer (UICC II/III) consists of neoadjuvant CRT, followed by standardised radical surgery including total mesorectal excision (TME) [4–8]. While this treatment results in improved oncological control, surgical resection may have severe complications, including anastomic leakage, which occurs in 10–20% of patients [9–11], accompanied by increased morbidity and mortality. Furthermore, many patients are suffering from the side effects of surgery: up to 27% of patients need a permanent stoma [12], 39% experience faecal incontinence [13, 14], 30% urinary [15] and 67% sexual dysfunctions [16, 17], all resulting in a severe reduction in quality of life.

The response to preoperative CRT is very heterogeneous, ranging from a pathological complete response (pCR) with no viable tumour cells left (10–25% of patients) to virtually no tumour regression at all or even tumour progression during therapy [5, 18, 19]. Patients with a pronounced tumour regression have a favourable oncological outcome, with a 5-year overall survival probability of more than 90% [20]. Importantly, residual lymph node metastases, even after complete regression of the primary tumour, indicate a poor prognosis [21, 22]. In 2004, Habr-Gamma et al. presented data of a selected group of rectal cancer patients, assessed to be clinically complete responders (cCR) and consecutively spared from surgery (“*watch and wait*”). These patients had a similar oncological outcome compared to standard treatment [23]. Forgoing surgery spares patients with a cCR from operation associated morbidity and mortality as later shown by Maas et al. [18, 24]. Conversely, as retrospectively collected data revealed an adverse outcome for “watch and wait” patients compared to the initial report of Habr-Gama et al. [25], this concept was rejected by a majority of surgeons. It was later shown that the data were too heterogeneous, possibly due to an incorrect assessment of cCR. This emphasises the importance of a stringent assessment of cCR as the key to the acceptance of a “watch and wait” strategy. Currently the selection of patients is imaging-based and incorporates clinical and endoscopic parameters. Nevertheless, the rate of local recurrence within the first two years is ~25%, as recently published by the international “watch and wait” consortium [26]. Molecular markers, as outlined by Yoo et al. [27], could augment the development of pretreatment response-prediction models improving the selection of patients for a “watch and wait” strategy.

This dilemma precipitated a plethora of studies trying to predict response to CRT by gene-expression profiling of pretreatment biopsies. However, efforts to establish universal molecular response predictors were thus far not successful [28, 29] because the validation of proposed predictors in independent datasets mostly failed. Consequently, none of these classifiers ever translated into routine clinical practice.

Here we present data from gene-expression profiles of pretreatment rectal cancer biopsies from 161 patients that were treated within the CAO/ARO/AIO-94 and CAO/ARO/AIO-04 trials using a machine learning approach to build a classifier predicting pCR to preoperative CRT. The classifier was validated in three independent cohorts (*n* = 76).

## Materials and methods

### Patients, clinical staging and treatment

Pretreatment biopsies were collected during the initial staging endoscopy between 2001 and 2012 at six surgical departments, all members of the “*German Rectal Cancer Study Group (GRCSG)”*. A total of 175 patients from a consecutive series of 236 patients with locally advanced rectal cancer (cUICC II/III) fulfilled inclusion requirements, regarding RNA quality, tumour cell content, complete clinical annotation and array quality (CONSORT Diagram, Fig. S1A). The study was approved by the local ethics committees, and all participants gave written informed consent. Detailed patient characteristics can be found in Table S1A. Precise staging and treatment procedures are described in Fig. S2 and in Supplementary Materials and Methods. Macroscopic and microscopic assessment of tumour stage was performed as previously described [4]. Residual tumour after CRT was assessed as a ratio of remaining viable tumour cells and the area covered by the tumour before treatment as described previously by Roedel et al. [5]. However, the tumour regression grading was reported in percent (TRG in %) to achieve a more accurate spectrum of response. Additionally, TRG was evaluated using the classification of Dworak et al. [30]. Pathological complete response (pCR) was defined as the absence of viable tumour cells in the primary tumour and lymph nodes (TRG = 100%, TRG 4, ypT0 pN0). Four to six weeks after surgery patients received an adjuvant therapy of either 5-FU alone or in combination with folinic acid and Oxaliplatin [4, 8].

For 14/175 patients treated at three German surgical centres we were unable to obtain TRG in %, so these cases were only used as the first independent validation dataset (Table S1B).

For the second independent validation dataset, clinical and gene-expression data of additional 38 patients with local advanced rectal cancer treated at the Department of Surgery, University of Padua, Italy, were acquired from the GEO database [31] (Table S1C).

For a third independent validation, a clinical trial was set up, named TransValid A (German Clinical Trial Register No 00003659). The goal of this open, non-randomised, prospective exploratory multicenter validation study was to collect biomaterial from patients with stage II/III rectal cancer treated according to established multimodal therapy. Inclusion criteria were adjusted to the previous trials of the GRCSG [7, 8]. Overall, 25 patients from 6 institutions were included. Applying the same quality criteria as for the first two sample sets 24 patients could be included in the final analysis (Fig. S1B and Table S1D).

### Pretreatment tumour biopsies, RNA isolation and gene-expression microarray analysis

Biopsies were collected during the initial staging rectoscopy and immediately stored in RNAlater as previously described [32, 33]. The tumour content of the biopsies was analysed by a pathologist, and only samples containing more than 50% of tumour tissue were used in this analysis. Gene-expression microarray (Human 4 × 44 K v2 gene-expression array, Agilent Technologies (G4845A)) analysis was performed per manufacturer’s instructions as previously described [34–36]. Gene-expression data were deposited to Gene Expression Omnibus (GSE87211). Details are described in Supplementary Methods.

### Statistical methods

Metric, ordinal and categorical parameters were compared between complete and partial responders by the two-sample *t*-, Mann–Whitney U- and Fisher’s exact test, respectively. Survival times were compared by Kaplan–Meier curves. Independent cohorts of Microarray gene-expression data were log-transformed and normalised using the quantile method. The normalisation algorithm was initially applied to the first dataset, and the quantiles of the validation datasets were then aligned to the same distribution. MATLAB R2018b Support Vector Machine functions were used to train and test classifiers. Performance was evaluated via area under the curve (AUC) resulting from receiver operating characteristic (ROC) curves. All programming work in this study was done in MATLAB 2018b (The MathWorks Inc., Natick, MA, 2000).

### Evaluation of classifiers

The classification performance was evaluated via two measures. The first is the area under the curve (AUC) of the receiver operating characteristic curves (ROC) of classification. The second is the maximal sensitivity (True positive rate) reached when the false-positive rate is zero (i.e. the maximal rate of true pCR captured when no patients were misclassified as pCR). For simplicity, this measure is termed sensitivity throughout the manuscript.

### Feature selection, cross-validation and support vector machine (SVM) classifier training

For feature selection, out of the 161 cases enrolled in this study, 32 positive (pCR, TRG = 100%) and 32 negative (poor response, TRG < 45%) cases were used. The samples were randomly divided 500 times into a training set (3/4 of the samples) and a test set (the remaining 1/4). Each time we performed a differential gene-expression analysis. A hill-climbing [37] procedure was applied to each of the resulting 500 gene sets to generate Support Vector Machines (SVMs). To evaluate the predictive power, each of the 500 resulting SVMs was applied to their corresponding test sets. Finally, genes that were selected a significant number of times (*P*-value < 0.05, Supp. Methods) as having optimal sensitivity on the test sets, i.e. a set achieving the highest percentage of correctly identified positive samples, while not allowing any false positives in the test set, were used as features for the classifier. With the resulting 21 transcripts, we performed a four-fold cross-validation. The full set of 64 samples and the classifier signature were used to train a final SVM which was applied to all 161 cases, with a tumour regression grade ranging from 10 to 100%. Subsequently the classifier was applied to independent datasets. A detailed description, as well as a graphic visualisation of the feature selection process, is shown in Fig. S4 and Supplementary Methods.

### Evaluating previously published signatures for predicting response to CRT

Gene-expression signatures from five studies, published by us and others [29, 38–41], that aimed to predict response to preoperative CRT, were analysed. The gene lists were adapted to unify annotation. The predictive performance of each signature was independently evaluated in our dataset of 161 patients and in a second dataset [31]. Therefore, a SVM classifier with a linear kernel was used via a leave-one-out cross-validation, as performed in these studies. The resulting performance (in terms of AUC) for each of these classifiers was established (Table 1). Additionally, a heatmap and PCA were plotted for each signature (Fig. S5).

**Table 1:** Comparison of the performance (in terms of AUC) of published gene signatures (GS) identifying pCR when applied to our primary patient cohort (*n* = 161) and the one from Millino et al. [31] (*n* = 38).

Test set	our signature	GS1	GS2	GS3	GS4	GS5
161 samples	0.81	0.51	0.55	0.61	0.27	0.59
38	0.76	0.48	0.68	0.63	0.74	0.4

GS1 was published by Lopes-Ramos et al. [29]; GS2 by Ghadimi et al. [39]; GS3 by Empuko et al. [40]; GS4 by Watanabe et al. [38] and GS5 by Kim et al. [41].

### Validation of our SVM classifier in three independent rectal cancer datasets

Independent dataset I: 14 patients, all treated within the CAO/ARO/AIO-94 or -04 study. These patients were not included in the initial analysis because we were unable to obtain the percentage of TRG. However, as TRG according to the classification of Dworak [30] was available, these patients were used as an independent validation set. Samples with TRG = 4 were considered pCR (*n* = 4).

Independent dataset II: Gene-expression and clinical data of 38 rectal cancer patients published by Millino et al. [31]. Samples with TRG = 1 per the classification of Mandard [42] were considered pCR (*n* = 8), patients with TRG > 1 were considered incomplete responders (*n* = 30). Additional patients published as a validation dataset by Millino et al. [31] were not analysed, as there was no annotation of pCR.

Independent dataset III (TransValid A trial): Additional pretreatment biopsies and clinical data were collected after 2012 and analysed prospectively on a day-to-day basis (*n* = 25). Four patients had a pCR (TRG = 100%), 20 were incomplete responders (TRG < 100%).

All gene-expression data were quantile-normalised using the mean expression values of the initial gene-expression data (*n* = 161) as reference distribution and the predictor was applied to the normalised gene-expression patterns.

### Building a second, not normalisation-dependent, score-classifier based on the 21 genes

SVM classifiers require a data distribution similar to their training set and are hence normalisation dependent. Normalisation introduces additional bias and requires trained personnel. To demonstrate the usefulness of the 21 genes, a second classifier, that does not require any transformation of the data, was trained. The second classifier is a score, based on the expression levels of the 21 genes selected for the SVM classifier. First, 64 cases, comprising 32 positive (pCR, TRG = 100%) and 32 negative (poor response, TRG < 45%) cases, were randomly divided into a training and test set with two-fold cross-validation. In 100 iterations, a simulated annealing procedure was used to train the score. The procedure starts with a random assignment of genes to the numerator and/or denominator of the score. In each step, all possible modifications of the score (additions or removals to or from the numerator or denominator) are evaluated. The score is adjusted in a way that maximises the sensitivity of the training (until reaching a maxima). The 100 trained scores are then evaluated in the test set. The score-classificator with maximal test sensitivity is selected:

$$\frac{{\mathop {\sum }\nolimits_{i = IAA1598,ASPM,TMPO,HOMER1,CXCL10,CENPL,BRCA1,FZD10,C19orf51,C20orf26,CASC5,CCNB1,FANCM,BLM} {{{{{\mathrm{Exp}}}}}}_i}}{{\mathop {\sum }\nolimits_{i = CGREF1,TNPO3,XPO1,TSNAX,CENPL,FANCM,FZD10,C19orf51,C20orf26,CCNB1,CSPP1,BLM} {{{{{\mathrm{Exp}}}}}}_i}}$$

Analog to the SVM-classifier, the final score-classificator is applied to all 161 cases (tumour regression grade ranging from 10 to 100%) and to three independent datasets comprising additional 78 patients. Additionally the score classifier was applied to the expression data of 28 patients from Canto et al. [43] (GSE123390), the resulting ROC curve can be found in the Supplementary Fig. SF7.

### Ingenuity pathway analysis (IPA)

The 21 transcripts comprising the classifier were uploaded into the IPA® software (Ingenuity, QIAGEN, Hilden, Germany), and a pathway, as well as a network analysis, was performed. Only experimentally validated edges were considered.

### Testing the predictive value of the classifier in rectal cancer patients

As a response to preoperative CRT is associated with a favourable outcome, we speculated that the classifier should also identify patients with a good clinical prognosis. Therefore, patient data from the “The Cancer Genome Atlas” (TCGA) were analysed. We used the classification scores obtained from applying the trained classifier to the gene-expression data of the 21-transcript used to train the classifiers (applied to the mRNA values, as is, using *RNA_Seq_v2_expression_median*). The classifier was applied to the 161 patients in our rectal cancer dataset, and the resulting classification scores were correlated with the TRG in percent of the patient are plotted.

Then, cancer survival and gene-expression data from 468 colon and rectal cancer patients were retrieved from the READ-TCGA [44], and the classifier was applied to this data as well. The top and bottom 50% of the resulting classification scores were used to plot the Kaplan–Meier survival curve assessing overall survival (OS), and disease-free survival (DFS).

## Results

### Patient characteristics

Patient characteristics for the test and training as well as the independent validation sets are summarised in Table S1A-C and Fig. S3. For neither dataset was there a significant correlation of response with age, sex, tumour stage and addition of oxaliplatin to CRT. There was also no significant (*p* = 0.346) distribution difference between responders and nonresponders according to their Consensus molecular subgroups (CMS) [45], see Table S1F.

### Identification of a gene-expression signature that predicts pathological complete response (pCR)

**Fig. 1:**
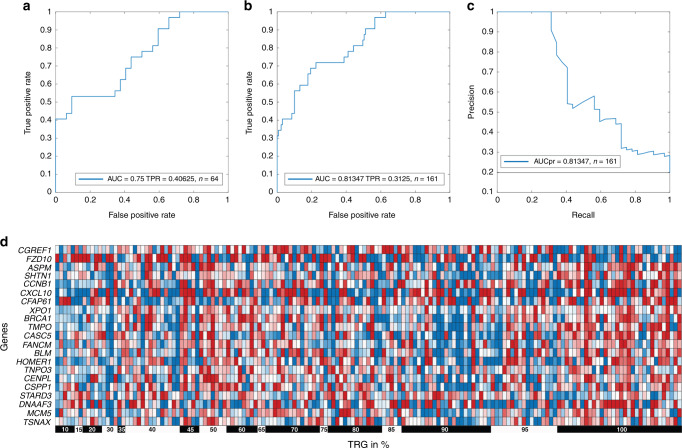
A 21-transcript signature robustly identifies complete responders. **a** Receiver Operating Characteristic (ROC) curve resulting from a four-fold cross-validation using 32 positive and 32 negative cases. The area under the curve (AUC) and the True positive rate (TPR) are reported. **b** ROC plot of the classifier when tested on 161 cases, with a full range of tumour regression grading in percent (TRG 10–100%) and **c** precision recall curve. **d** Heatmap visualisation of the expression of the 21-transcript signature in 161 pretreatment biopsies of rectal cancer patients.

As the purpose of this study is to reliably identify pathological complete response (pCR) without misclassifying incomplete responders (false-positive rate = 0), we performed feature selection aiming to maximise the performance of the classifier. Thirty-two positive (pCR, TRG = 100%) and 32 negative (poor response, TRG < 45%) cases were chosen for feature selection and generation of the classifier. These 64 cases were randomly divided into training and test sets. Differentially expressed genes between pCR patients and incomplete responders were identified in the training set. A hill-climbing feature selection [37] was applied, gradually adding genes/features, that improve the sensitivity of the resulting classifier when applied to the test set. The procedure of splitting 64 samples into two groups, performing differential gene-expression analysis and generating a classifier with maximal sensitivity when applied to the test set was repeated 500 times. A signature of 21 transcripts, that were consistently selected (*P*-value < 0.05) to have maximal sensitivity during the 500 repetitions, was chosen to generate a final classifier (Table 2). A four-fold cross-validation procedure on 32 positive and 32 negative cases resulted in a sensitivity of 0.4 and an AUC of 0.75 (Fig. 1a). Based on these 64 cases, an SVM approach was applied to all 161 cases. Encouragingly, applying our classifier to the entire dataset, it achieved a sensitivity of 0.31 and an AUC of 0.81 (Fig. 1b), indicating that even when considering the full range of TRG (10–100%), our SVM-classifier can correctly identify more than a third of complete responders without error. The tradeoff between precision and recall is visualised in Fig. 1c.

**Table 2:** List of 21 transcripts in the classifier.

Gene symbol	Probe	Systematic name	Chromosome	Probe sequence 3’–5’
*CGREF1*	A_33_P3281850	CR623121	2	GGTGGTCACTTCTAACTCGTCATTCACCAACAGCAGCCTAGCGTTGCCCCCATTACAACA
*FZD10*	A_23_P203972	NM_007197	12	CTTCACAGTGCCAGGAAAGAGTGGTTTCTGCGTGTGTATATTTGTAATATATGATATTTT
*ASPM*	A_23_P52017	NM_018136	1	ATCACAAATCCCCTGCAAGCTATTCAAATGGTGATGGATACGCTTGGCATTCCTTATTAG
*SHTN1*	A_23_P202587	NM_018330	10	GTAATAATTGCAGTAGTTGTATTGTATTGTATTTTTGCACGTGTGGTAAGCATAGGCTTG
*CCNB1*	A_23_P122197	NM_031966	5	GGACACCAACTCTACAACATTACCTGTCATATACTGAAGAATCTCTTCTTCCAGTTATGC
*CXCL10*	A_24_P303091	NM_001565	4	GTCAAGCCATAATTGTTCTTAGTTTGCAGTTACACTAAAAGGTGACCAATGATGGTCACC
*CFAP61*	A_32_P4262	NM_015585	12	TTTACAAATCCCAGGCATGTTCCTGTGCAGAATAATCCATTTCAGCAATAAAATGAGATC
*XPO1*	A_23_P40078	NM_003400	10	GGGTATTTGTCGACCAAAATGATGCCAATTTGTAAATTAAAATGTCACCTAGTGGCCCTT
*BRCA1*	A_23_P207400	NM_007300	17	AAGTGTTTTTCATAAACCCATTATCCAGGACTGTTTATAGCTGTTGGAAGGACTAGGTCT
*TMPO*	A_23_P325040	NM_003276	12	AAAGTAATTGCCTGTGTAGAACTACTTGTCTTTTCTAAAGATTTGCGTAGATAGGAAGCC
*CASC5*	A_23_P100127	NM_170589	15	CGGTCTCTAGCAAAGATTCAGGCATTGGATCTGTTGCAGGTAAACTGAACCTAAGTCCTT
*FANCM*	A_32_P106732	NM_020937	14	AATCAAGCTGCTCAAGATGGGGTTTTCAAAGACCTCTCACAATATTAAATGCACTTCAAT
*BLM*	A_23_P88630	NM_000057	15	TATGCATTCTCATAACAACCGAATCTCAATGTACATAGACCCTCTTTCTTGTTTGTCAGC
*HOMER1*	A_33_P3372257	NM_004272	5	GAGCACCAAGTTTTAATTTAAATAGGAGATTTAACACTAGGGATCAGGGAGTTTAGTATG
*TNPO3*	A_33_P3370132	NM_012470	7	TAAAAGGTTTGCCAAAGGAAACAACCGTGGGAGCCGTCACAGTGACACACAAACAACTTA
*CENPL*	A_23_P126120	NM_033319	1	TTTCAAAATTCATTTATCAGCCACAAGATTAGTTCGTGTTTCAACATCTGTAGCTTCAGC
*CSPP1*	A_23_P71537	NM_001077204	8	TGGTTGACCCTGATGACATCATGAAACACATAGGGGATGACGGATCAAACTCTGTAGCAA
*STARD3*	A_33_P3246804	NM_001165937	17	CCTGTCACCCGTGTGAAGATGAAGGGGCTCTTCATCTGCCTGCGCTCTCGTCGGTTTTTT
*DNAAF3*	A_33_P3286349	NM_178837	19	ATTGTTCTCTCAGAATTCCAAATTCCACTTCTGAGGCTCTAAGCCCAGCCTAGGATCTGA
*MCM5*	A_23_P132277	NM_006739	22	CAGCATCATCAAGGACTTCACCAAGCAGAAATACCCGGAGCACGCCATCCACAAGGTGCT
*TSNAX*	A_24_P148151	NM_005999	1	AAAGTTGAGTTATATACTTGTACATACAATGGAAATGCTTTTAGTAGTGATTATTTAGCA

### Previously published signatures are poor predictors of response in independent datasets

A major issue impeding the emergence of robust prognostic or diagnostic tools from molecular signatures is the poor reproducibility in independent datasets [46]. We therefore compared our SVM-classifier with classifiers published earlier by us and others [29, 38–41] on our dataset of 161 patients as well as on the 38 patients published by Millino et al. [31]. As shown in Table 1 and visualised in Fig. S5, none of the published signatures was useful for response prediction.

### The 21-transcript classifier robustly identifies over 1/3 of patients with pCR in independent datasets

**Fig. 2:**
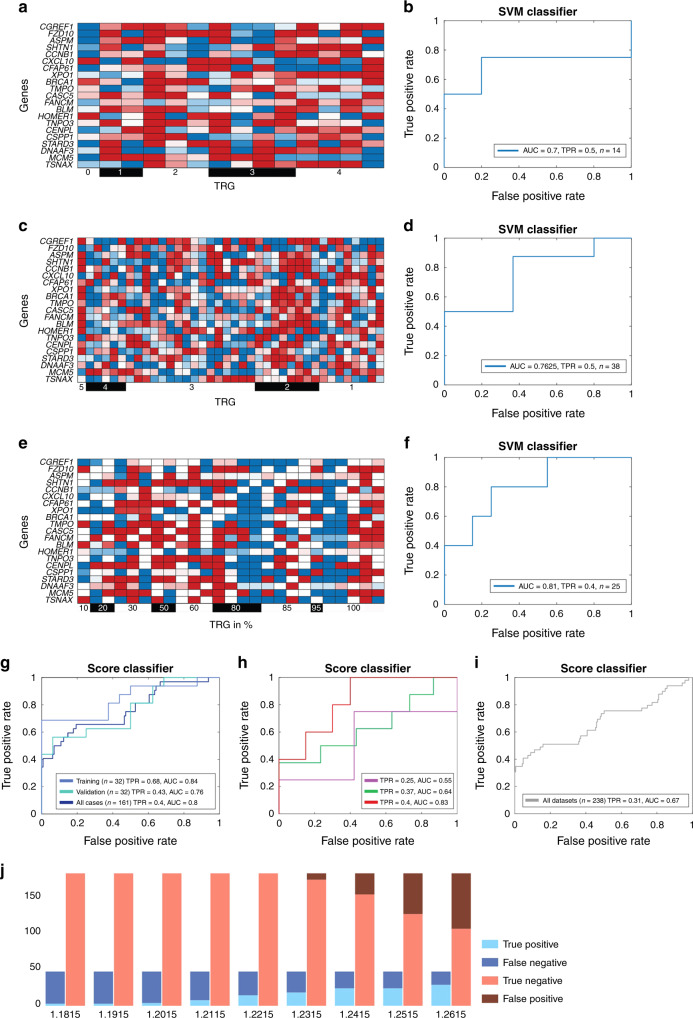
The SVM classifier and the score classifier robustly predict pathological complete response in three independent patient cohorts, while never misclassifying a partial responder as complete. **a** Heatmap visualisation and **b** ROC curve of the SVM-classifier performance 14 patients from the CAO/ARO/AIO-94 or 04 trial, not examined in the first analysis. Two out of 4 patients with pCR were identified. **c** Heatmap visualisation and **d** ROC curve of SVM-classifier performance for 38 patients published by Millino et al. [31]. Four out of 8 patients with pCR were identified. **e** Visualisation and **f** ROC curve of SVM-classifier performance when applied to 25 prospectively collected specimens from the TransValidA trial. Two out of 5 patients with pCR were identified. **g** The score-classifier performance for the training, validation and all 161 cases, with a full range of tumour regression grading in percent. **h** ROC curve of the score-classifier performance for 14 patients from the CAO/ARO/AIO-94 or 04 trial (purple, one out of 4 patients with pCR were identified), for 38 patients published by Millino et al. [31] (three out of 8 patients with pCR were identified) and for the 25 prospectively collected specimens from the TransValidA trial (red, two out of 5 patients with pCR were identified). **i** ROC curve of the score-classifier performance for the integration of all 238 samples in this study. **j** Bar plots showing the prediction accuracy and error types for different score thresholds on the aggregate compendium of the 238 patients included in all datasets studied (161 + 14 patients from the CAO/ARO/AIO-94 or -04 trial, 38 cases published by Millino et al. [31], 25 patients included in the TransValidA trial).

To test if our SVM-classifier can identify patients with pCR in independent datasets we applied it without any further training, to three additional patient cohorts. The first one comprised 14 patients treated within the CAO/ARO/AIO-94 or 04 studies. The classifier identified two out of four patients with pCR (Sensitivity = 0. 5, AUC = 0.7), while none of the patients was wrongly classified as pCR (Fig. 2a, b). In a dataset of 38 patients (8 pCR), recently published by Millino et al. [31], the classifier correctly identified four patients to have pCR (Sensitivity = 0.5, AUC = 0.76), again not misclassifying any patient incorrectly as pCR (Fig. 2c, d). Finally, the classifier was applied to 25 prospectively collected patients and could correctly identify two out of five patients with pCR (Sensitivity = 0.4 AUC = 0.81), again not misclassifying any patient as pCR when they were not (Fig. 2e, f). A graphic illustration of the support vector values used for the SVM-classifier can be found in Fig. S6 and the full support vector is provided in Table S2. The classification was independent of demographic parameters (Fig. S3).

To further demonstrate the clinical relevance of the 21 genes, we developed a second, simple, score-based classifier using the 21 genes (Methods, Fig. 2g). This simple classifier does not require normalisation yet allowed good identification of a considerable percentage of the pCR patients, while not misclassifying any patient incorrectly as pCR (Fig. 2h). It identified more than 30% of all pCR samples in the independent datasets, without misclassifying any patient incorrectly as pCR (Fig. 2i, j).

### Expression of the 21-transcript score correlates with treatment response and survival of colorectal cancer patients

**Fig. 3:**
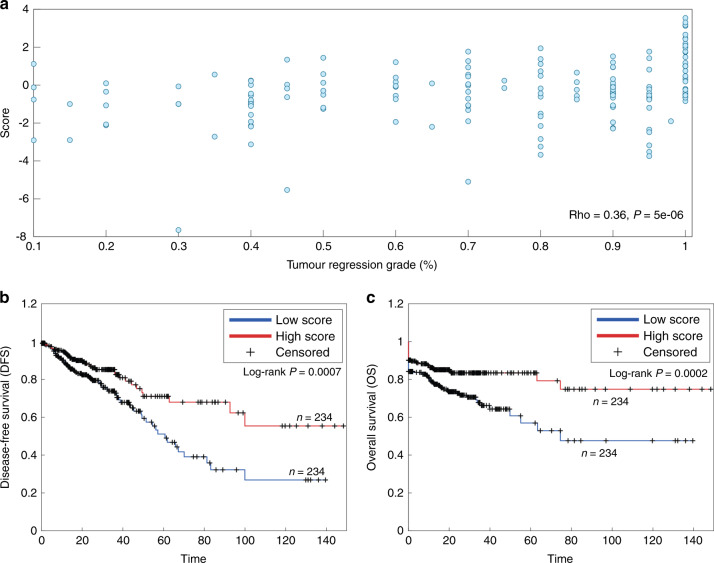
The classifier score based on the expression of the 21 transcripts correlates with TRG and is associated with outcome in an independent dataset. **a** Correlation of the classifier score and TRG in 161 rectal cancer patients. **b** Kaplan–Meier curves of colon and rectal cancer patients from the TCGA (*n* = 468) comparing overall survival (OS) of 50% of patients with the highest vs. 50% with the lowest classifier score. **c** Kaplan–Meier curves of colon and rectal cancer patients from the TCGA (*n* = 468) comparing disease-free survival (DFS) of 50% of patients with the highest vs. 50% with the lowest classifier score.

pCR is associated with improved survival and a more favourable oncological outcome [18, 47]. We therefore hypothesised that our classifier would also be useful for disease prognostication. To this end, we first used the classifier score based on the expression of the 21 transcripts on the 161 rectal cancer patients. As expected, we observed a significant (Spearman *rho* = 0.36, *p* = 5e-06) correlation with tumour regression (Fig. 3a). We then applied the classifier to 468 colon and rectal cancer patients from the TCGA database to obtain classification scores and found that high classification scores were significantly (*p* = 0.0007) associated with longer overall survival (Fig. 3b) and a significantly (*p* = 0.0002) longer disease-free survival (Fig. 3c).

### Ingenuity pathway analysis (IPA) identifies enrichment of radiation response pathway genes

To gain insight into the biological mechanisms of the 21 transcripts comprising the classifier an IPA®-pathway analysis was performed. There was significant enrichment for genes involved in DNA-damage control pathways; the “*Role of BRCA1 in DNA Damage Response”* (*p* = 0.002239) pathway, “*DNA G2/M DNA Damage Checkpoint regulation”* (*p* = 0.012589) and “*ATM signaling”* (*p* = 0.002239) were, after correction for multiple testing, significantly over-represented (Table S3).

## Discussion

Sparing rectal cancer patients from severe side effects and complications associated with radical surgery or even amputation (removal of the anus, the rectum, partly the sigmoid colon and permanent colostomy) is an attractive option for patients with a cCR after preoperative CRT. Unfortunately, the number of local relapses is still high, indicating shortcomings in the initial assessment of cCR which requires patients to undergo a strictly standardised follow-up, including frequent clinical, endoscopic and image-based surveillance, in highly specialised centres [26]. Even though the majority of patients with tumour regrowth can be salvaged with definite surgery after “watch and wait” [48], there is an urgent clinical need for additional parameters to correctly identify complete responders.

Recent clinical trials in other tumour entities demonstrated that gene-expression profiling can be useful. Examples are the MammaPrint [49, 50] signature for breast cancer, the OncoTypeDX® [51] and ColoPrint® for colorectal cancer [52, 53].

Exploring the utility of gene-expression profiling for the prediction of response to preoperative CRT in rectal cancers has been actively pursued. However, despite several studies reporting promising results, none has translated into a clinical test to guide individualised therapy [54, 55]. Two studies tried to identify markers that predict pCR in rectal cancer. The first, published by Kim et al. [41], using gene-expression profiling of pretreatment (*n* = 31/11 pCR) biopsies to build a classifier. When applied to an independent dataset (*n* = 15/4 pCR), the signature identified all four pCR patients correctly, but also identified two incomplete responders as pCR. The second study was performed by Brettingham-Moore, analyzing 51 cases (7 pCR). However, they could not identify reproducible predictive signatures [28].

Since previously published gene-expression signatures for response prediction lacked reproducibility [29], we re-tested several of these classifiers and could also not observe a reliable prediction of response in our patient cohort (Table 1, Fig. S5). A possible explanation for this is that feature selection was performed in parallel to the training procedure, which increases the risk of overfitting [56, 57]. Our model was initially designed to discriminate between the extremes in response to CRT (pCR vs poor response), but it remained robust when applied to the full range (0–100%) of response (Fig. 1). It identifies over 1/3 of the patients with pCR in all datasets examined and, most importantly, when considering a possible clinical application, never classifies any patient with an incomplete response as pCR. Unlike previous studies we aimed to predict pCR with the highest accuracy, at the cost of sensitivity [58]. Allowing mistakes when training a classifier would increase the rate of identifying patients with pCR; however, the clinical benefit of such a predictor would be limited. Finally, unlike previous studies, we show that using the same 21-transcript signature in independent, comparable datasets previously published by others (Fig. 2c, d), robustly classifies pCR, which is essential for clinical applicability. Additionally, we show that our classifier predicts response in routinely collected samples: for the TransValid trial we did not collect a set of pretreatment biopsies and analysed them in one batch. For every four patients, RNA was isolated and gene-expression levels analysed. This method introduced additional bias, but resembles the infrastructure of routine day-to-day clinical testing (Fig. 2e, f). To overcome these issues, the test microarray datasets were quantile-normalised before applying the SVM classifier, which introduces a limitation to the approach which is, necessarily, dependent upon normalisation.

To demonstrate that the 21 gene signature is also useful without any data transformation, we trained an additional classifier. The second classification score was developed using the same 21 genes, it does not require normalisation and can be applied to any data as is (Fig. 2g–j). Both classifiers robustly predicted pCR in all test datasets. Even when applied to additional independent data, published by Canto et al. [43], missing values for some of the 21 genes, the score classifier stayed robust (Supplementary Fig. SF7) underscoring a potential clinical utility.

Investigating the cases that were not identified as pCR in our data by the two classifiers, we did not identify genes that were up or downregulated in these samples. We additionally did not identify differences in stage or age between the correctly classified and misclassified samples, but we did find that the misclassified samples included more female patients (46%, compared to 32% in the total set of patients). It is possible that the performance for female patients is impaired because the classifier was trained using predominantly male patient samples (as a result of the increased frequency of rectal cancer in male compare to female patients). Future studies are therefore warranted to thoroughly investigate the need for sex-balanced patient cohorts when training pCR classifiers.

To further strengthen the potency of the classifier we used it as a predictor of prognosis. Knowing that pCR is associated with a good prognosis determined by disease-free survival and overall survival [18, 59], we speculated that patients with a high score based on the classifier genes should also have a favourable outcome. As the follow-up data for our 161 patients is relatively short, and to test our classifier in another independent patient cohort, we applied it to 468 colon and rectal cancer patients from the TCGA database. Tumour regression for these patients is not reported; we therefore used overall survival as a surrogate for pCR. We found that the 50% of patients with the highest “pCR score”, as determined by the expression of the 21 genes, had an excellent outcome (Fig. 3). This shows the clinical utility of the classifier in general, even though it was trained for the specific task of pCR prediction.

From a mechanistic point of view, the classifier contains, intuitively, genes associated with DNA-Damage control and repair, underscoring the relevance of the signature. Most prominent are *BRCA1*, *BLM* and *FANCM*, all associated with the same DNA-damage response signaling pathway. *CCBN1* (Cyclin B1) and other genes are associated with cell cycle control, ATM signaling and DNA-damage checkpoint regulation. Furthermore, the classifier includes *FZD10*, a gene coding for one of the Frizzled receptors, activating the canonical Wnt/beta catenin pathway. Recently, we and others provided experimental proof that the Wnt-pathway mediates treatment resistance in rectal cancer [60–62]. Other genes are involved in response to cellular stress and other pathways associated with cellular survival. We assume that genes in the classifier might be suitable targets for overcoming treatment resistance.

Currently, watch and wait strategies are validated in clinical trials such as the ACO/ARO/AIO-18.1 randomised trial (NCT04246684). According to the International Watch & Wait Database (IWWD), local regrowth is expected in up to 25% of patients with clinical complete response (cCR) [63], indicating that a non-surgical approach for all cCR patients diagnosed on the basis of radiological and clinical data is unsupportable. Therefore, the identified signature and other molecular markers associated with treatment response, e.g. *TP53* mutations [64], or analysis of the tumour secretome [43], should be added to the decision making and validated in clinical trials [22]. Our data and the complex situation of neoadjuvant therapy do not support the reverse conclusion that non-pCR patients should be spared from preoperative CRT. We are currently exploring the conversion of the classifier to a NanoString® platform, which has been shown to robustly quantify gene-expression levels in routinely collected, formalin-fixed paraffin-embedded tissue.

In summary, we describe a 21-transcript signature that robustly predicts a pathological complete response in rectal cancer patients based on gene-expression profiles of pretreatment biopsies.

## Supplementary information

Supplemental Methods and Material

AJ-Checklist

## Data Availability

Gene-expression data were deposited to Gene Expression Omnibus (GSE87211).
