# Supplementary material for: Gene-expression profiles of pretreatment biopsies predict complete response of rectal cancer patients to preoperative chemoradiotherapy
Source: Br J Cancer. 2022 May 21;127(4):766–75. doi: 10.1038/s41416-022-01842-2 (PMC9381580; doi:10.1038/s41416-022-01842-2)
Supplement: Supplementary file 1 — Supplemental Methods and Material [file 41416_2022_1842_MOESM1_ESM.docx]

**Supplementary Data**

**Materials and Methods**

**Detailed staging and treatment procedures CAO/ARO/AIO 94 and 04**

Pretreatment staging included rigid rectoscopy and endorectal ultrasonography, magnetic resonance imaging (MRI) and/or computed tomography as well as histo-pathological diagnosis of an adenocarcinoma. Staging results were described as clinically assessed T-level (cT), lymph node status (cN), distant metastases (cM), and UICC stage (cUICC). Preoperative CRT was applied by a total irradiation dose of 50.4 Gray (28 x 1.8 Gray) and accompanied by either 5-fluorouracil infusion alone (n=88) or in combination with oxaliplatin (n=73), see Figure S2. Six weeks after the completion of preoperative CRT, curative total mesorectal excision (TME) surgery was performed.

**Pretreatment tumor biopsies, RNA isolation and Gene expression microarray analysis**

Biopsies were collected during the initial staging rectoscopy and immediately stored in RNAlater as previously described (1,2). Tumor content of the biopsies was analyzed by a pathologist and only samples containing more than 50 % of tumor tissue were used in this analysis. RNA was isolated using TRIZOL® and spin-columns (Qiagen, Hilden, Germany) as previously described (3), and sample quality was assessed using a BioAnalyzer; samples with an RNA Integrity Number (RIN) of < 5 were excluded (4) (Agilent Technologies, Santa Clara, CA, USA).

Gene expression microarray analysis was performed per manufacturer’s instructions as previously described (3,5,6). In brief, using the "Low RNA Input Linear Amplification Kit Plus, One Color" protocol (Agilent, Cat. N°: 5188-5339) 200 ng of total RNA was amplified and transcribed into fluorescence (Cy3) labeled cRNA which was subsequently hybridized to a Human 4 × 44 K v2 gene expression array from Agilent Technologies (G4845A). Fluorescence intensities were measured using an Agilent DNA microarray scanner (G2505B) at 5-micron resolution. Array images were analyzed by the Agilent feature extraction software. Signal intensities from the arrays were first log2 transformed and then normalized to the 75 percentile of overall signal intensity. Probes with maximum intensity over all samples of at least 100 were used for further analysis. Gene expression data were deposited to Gene Expression Omnibus (GSE87211).

**Feature selection**

For feature selection, out of the 161 cases enrolled in this study, 32 positive (pCR, TRG = 100%) and 32 negative (poor response, TRG < 45%) cases were used. The performance of the classifier was defined as the maximal True Positive Rate (TPR) when the *False Positive Rate* is zero. Aiming to maximize the performance of the classifier, the following prodedure was then repeated 500 times:

1. The 64 samples were randomly partitioned into a training set (¾ of the data, i.e. 48 cases with 24 positive/negative samples) and a test set (1/4 of the data, 16 cases with 8 positive/negative samples)

2. A differential expression analysis was applied to the training set using rank-sum test. The resulting group of genes differentially expressed between the positive and negative training set groups with rank-sum P-value<0.05.

3. A hill climbing stepwise feature selection(7) was applied, starting with an empty group of features; The addition of each gene from the differentially expressed genes was attempted for the construction of a Support Vector Machine (SVM) classifier using the training set, and the performance of the machine, when applied to the test set was evaluated. In each step, the gene that maximized the performace on the test set was added to the features group. The procedure terminated when no feature addition was inproving the performance.

Evantually, the features (genes) that were selected significant number of times (Binomial P-value<0.05) were selected as the final group of 21 feaures.

**Cross validation and classifier training**

After a signature of 21 genes has been established, a four-fold cross validation was performed to the 32 positive and 32 negative cases, each time leaving ¼ of these cases (with equal number of positive and negative class samples), training an SVM with the ¾ current training set, and applying it to the left out fold. The AUC and sensitivity resulting from the 4-fold cross validation are the training performace.

Then, the final classifier was trained using all 64 positive and negative samples. This classifier has been subsequently applied, as is, to the set of 161 samples with full range of TRG, and to all independent datasets.

**Supplementary Figures**

**SF1: CONSORT Diagram**


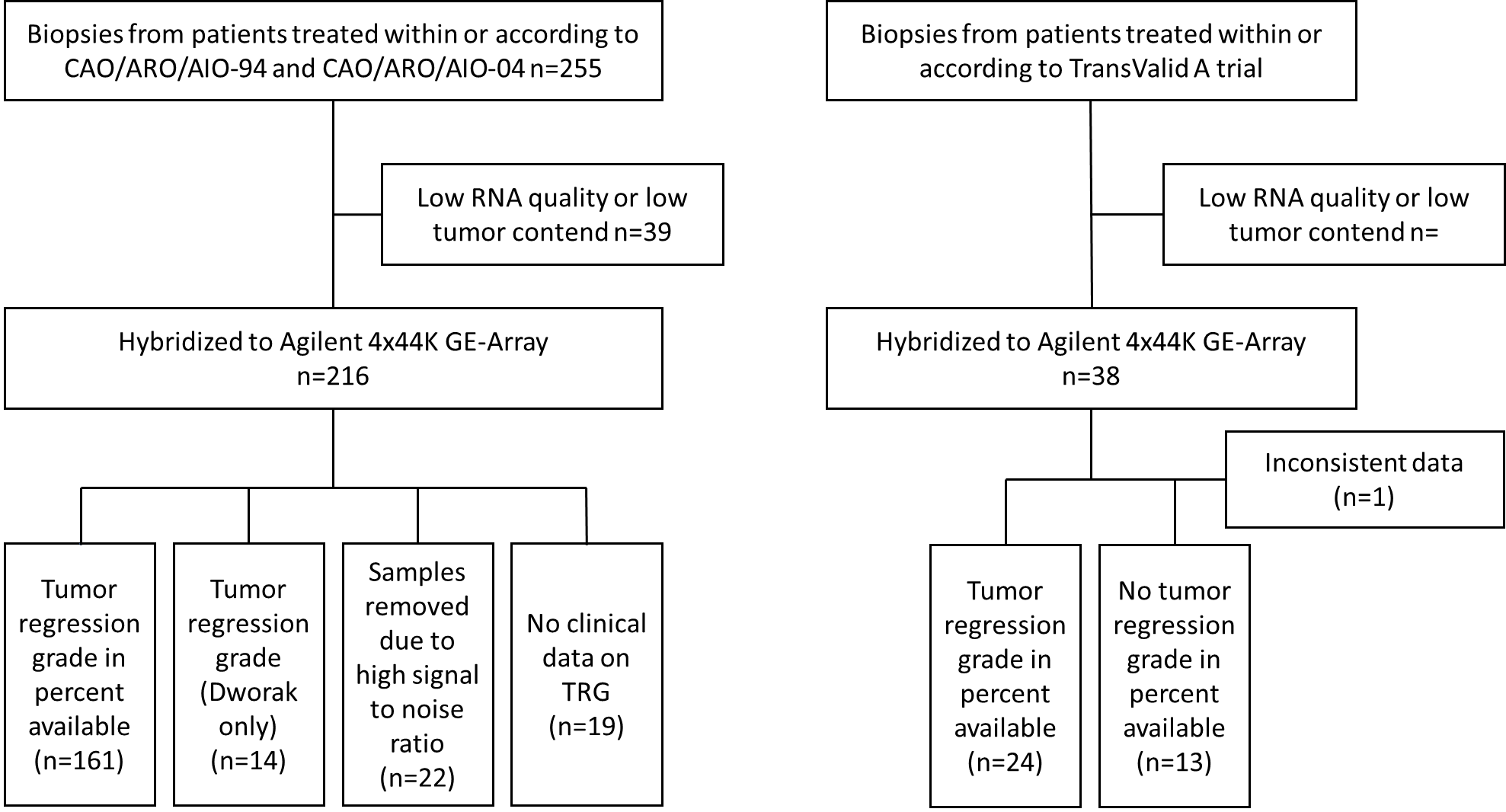


A

B

Supplementary Figure 1. Consort diagram of the participants from clinical trials.

**SF2: Schematic overview**

**
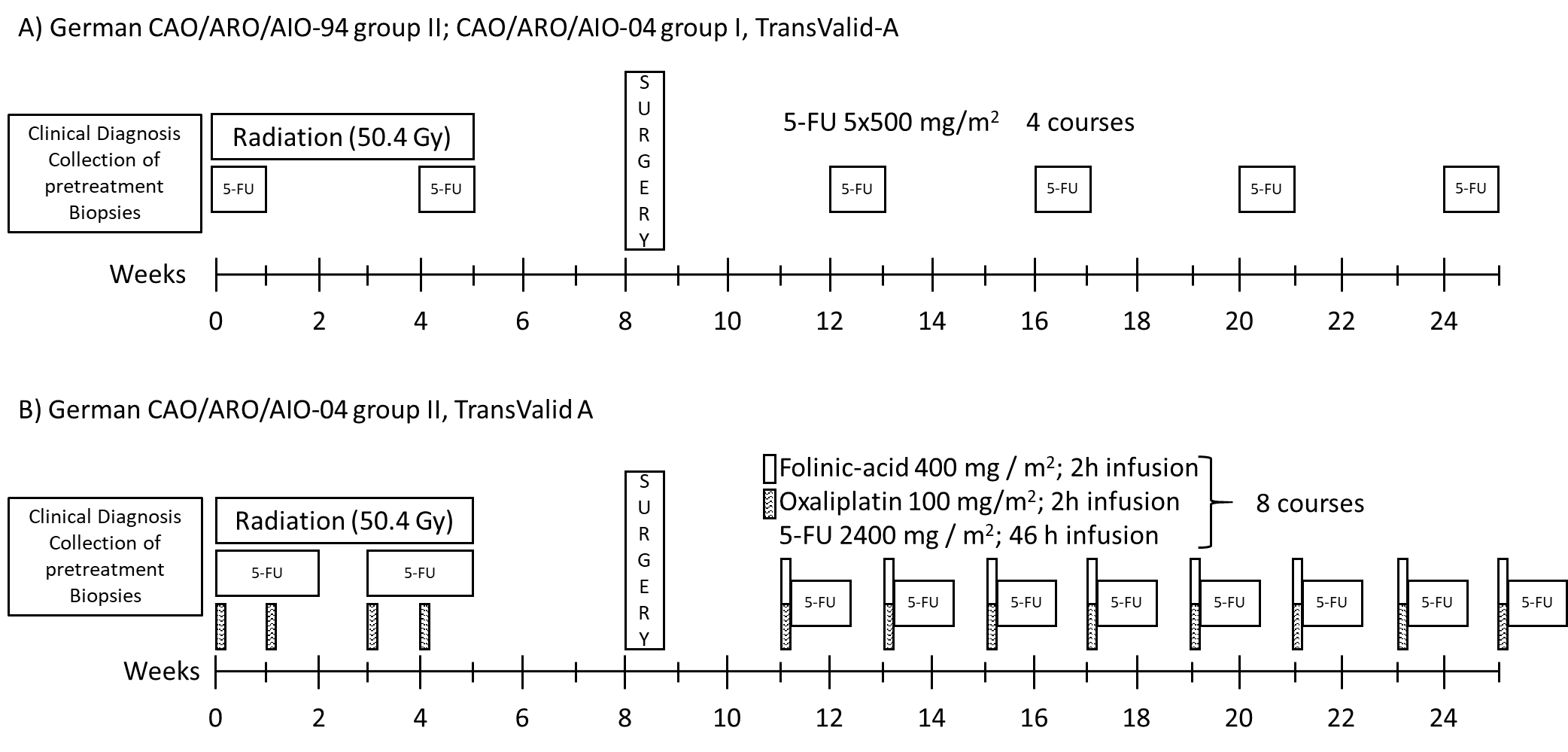
**

Supplementary Figure 2. Schematic overview of treatment concepts of the clinical trials.

**SF3: Patient Characteristics**

**
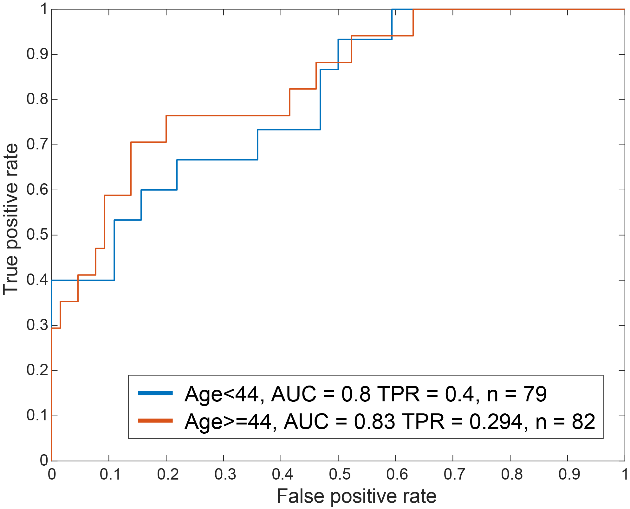

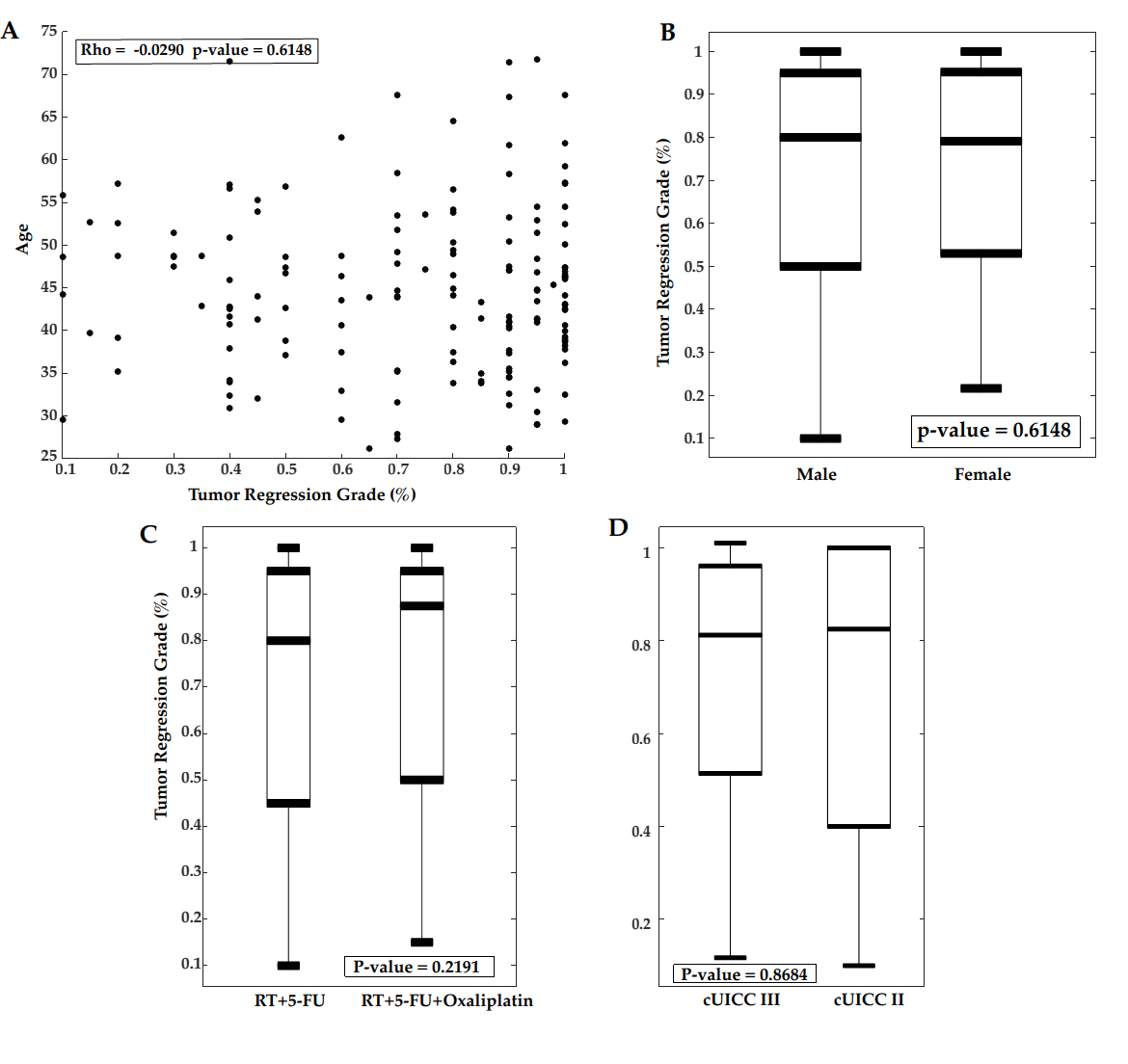
**

**E**

Supplementary Figure 3. A) Correlation of patient age and TRG. B Correlation of TRG and patient sex. C) Correlation of TRG and treatment modality. D) Correlation of TRG and initial tumor stage. E) SVM classifier performance in different age groups.

**SF4: Classification process pipeline**

Supplementary Figure 4. Classifier development pipeline.

**SF5: Visualization of previously published classifier performance**

GS1

GS2

GS3

GS4

GS5

Supplementary Figure 5. Heatmap visualization and PCA analysis of published gene signatures (GS) when applied to our primary patient cohort (n=161) (A,B) and the one from Milino et al. (8) (n=38) (B,C). GS1 was published by Lopes-Ramos et al. (9), GS2 by Ghadimi et al. (10), GS3 by Empuko et al. (11), GS4 by Wanranabe et al. (12) and GS5 from Kim et al. (13).

**SF6: Graphic illustration of the support vector values used for the SVM-classifier**


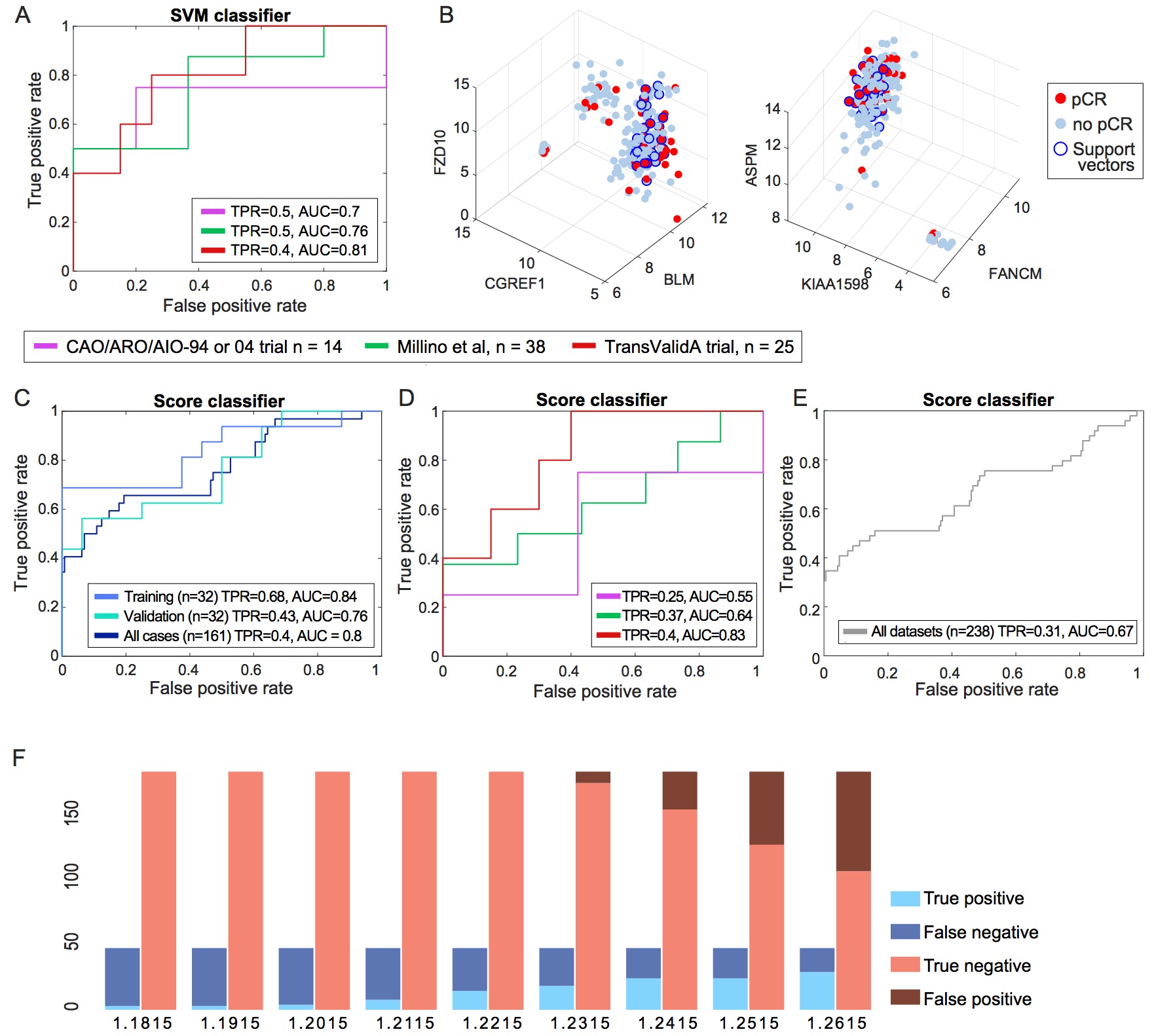


**SF7: ROC curve of the score-classifier performance for 28 patients from Canto et al (GSE123390)**

**Supplementary Tables**

**ST1. Patient information:**

**A)**

**B)**

**C)**

**D)**

**E)**

**F)**

|  | **pCR** | **non_pCR** | **total** |
| --- | --- | --- | --- |
| **CMS1** | 2 | 3 | 5 |
| **CMS2** | 3 | 14 | 17 |
| **CMS3** | 9 | 21 | 30 |
| **CMS4** | 7 | 33 | 40 |
| **NA** | 11 | 58 | 69 |
| **Total** | 32 | 129 | 161 |

Distibrution of cases according to the Consensus molecular subgroups (CMS). There was no significant difference in distribution (fisher exact test p value is 0.346).

**ST2. Support Vectors:**

**ST3. Deregulated Pathways:**

**References:**

1. Gaedcke J, Grade M, Camps J, Sokilde R, Kaczkowski B, Schetter AJ*, et al.* The rectal cancer microRNAome--microRNA expression in rectal cancer and matched normal mucosa. Clin Cancer Res **2012**;18(18):4919-30 doi 10.1158/1078-0432.CCR-12-0016.

2. Jo P, Nietert M, Gusky L, Kitz J, Conradi LC, Muller-Dornieden A*, et al.* Neoadjuvant Therapy in Rectal Cancer - Biobanking of Preoperative Tumor Biopsies. Sci Rep **2016**;6:35589 doi 10.1038/srep35589.

3. Grade M, Hummon AB, Camps J, Emons G, Spitzner M, Gaedcke J*, et al.* A genomic strategy for the functional validation of colorectal cancer genes identifies potential therapeutic targets. Int J Cancer **2011**;128(5):1069-79 doi 10.1002/ijc.25453.

4. Opitz L, Salinas-Riester G, Grade M, Jung K, Jo P, Emons G*, et al.* Impact of RNA degradation on gene expression profiling. BMC Med Genomics **2010**;3:36 doi 10.1186/1755-8794-3-36.

5. Camps J, Pitt JJ, Emons G, Hummon AB, Case CM, Grade M*, et al.* Genetic amplification of the NOTCH modulator LNX2 upregulates the WNT/beta-catenin pathway in colorectal cancer. Cancer Res **2013**;73(6):2003-13 doi 10.1158/0008-5472.CAN-12-3159.

6. Ghadimi BM, Jo P. Cancer Gene Profiling for Response Prediction. Methods Mol Biol **2016**;1381:163-79 doi 10.1007/978-1-4939-3204-7_9.

7. Kohavi R, John GH. Wrappers for feature subset selection. Artif Intell **1997**;97(1-2):273-324 doi Doi 10.1016/S0004-3702(97)00043-X.

8. Millino C, Maretto I, Pacchioni B, Digito M, De Paoli A, Canzonieri V*, et al.* Gene and MicroRNA Expression Are Predictive of Tumor Response in Rectal Adenocarcinoma Patients Treated With Preoperative Chemoradiotherapy. J Cell Physiol **2017**;232(2):426-35 doi 10.1002/jcp.25441.

9. Lopes-Ramos C, Koyama FC, Habr-Gama A, Salim AC, Bettoni F, Asprino PF*, et al.* Comprehensive evaluation of the effectiveness of gene expression signatures to predict complete response to neoadjuvant chemoradiotherapy and guide surgical intervention in rectal cancer. Cancer Genet **2015**;208(6):319-26 doi 10.1016/j.cancergen.2015.03.010.

10. Ghadimi BM, Grade M, Difilippantonio MJ, Varma S, Simon R, Montagna C*, et al.* Effectiveness of gene expression profiling for response prediction of rectal adenocarcinomas to preoperative chemoradiotherapy. J Clin Oncol **2005**;23(9):1826-38 doi 10.1200/JCO.2005.00.406.

11. Empuku S, Nakajima K, Akagi T, Kaneko K, Hijiya N, Etoh T*, et al.* An 80-gene set to predict response to preoperative chemoradiotherapy for rectal cancer by principle component analysis. Mol Clin Oncol **2016**;4(5):733-9 doi 10.3892/mco.2016.806.

12. Watanabe T, Komuro Y, Kiyomatsu T, Kanazawa T, Kazama Y, Tanaka J*, et al.* Prediction of sensitivity of rectal cancer cells in response to preoperative radiotherapy by DNA microarray analysis of gene expression profiles. Cancer Res **2006**;66(7):3370-4 doi 10.1158/0008-5472.CAN-05-3834.

13. Kim IJ, Lim SB, Kang HC, Chang HJ, Ahn SA, Park HW*, et al.* Microarray gene expression profiling for predicting complete response to preoperative chemoradiotherapy in patients with advanced rectal cancer. Dis Colon Rectum **2007**;50(9):1342-53 doi 10.1007/s10350-007-277-7.
